# Antiprotozoal Activity of *Artemisia vulgaris* and *Berberis vulgaris* Against *Leishmania major* and *Trichomonas vaginalis*

**DOI:** 10.1007/s11686-026-01224-1

**Published:** 2026-02-16

**Authors:** Tülay Aksoy, Nogay Girginkardeşler, İbrahim Cüneyt Balcıoğlu, Ali Ahmet Kilimcioğlu

**Affiliations:** 1 Department of Medical Parasitology, Faculty of Medicine, Inonu University, Malatya, Turkey; 2 Department of Medical Parasitology, Faculty of Medicine, Manisa Celal Bayar University, Manisa, Turkey

**Keywords:** Antileishmanial activity, Antitrichomonal activity, *Artemisia vulgaris*, *Berberis vulgaris*, *Leishmania major*, *Trichomonas vaginalis*

## Abstract

**Purpose:**

*Leishmania major* and *Trichomonas vaginalis* infections pose a significant global health burden, while current treatments are limited by toxicity, resistance, and restricted accessibility. This study aimed to evaluate the in vitro and ex vivo antileishmanial effects of *Artemisia vulgaris* and *Berberis vulgaris* extracts against *L. major*, as well as their in vitro antitrichomonal activity against *T. vaginalis* trophozoites.

**Methods:**

Ethanolic extracts of *A. vulgaris* and *B. vulgaris* were tested against *L. major* promastigotes, intracellular amastigotes, and *T. vaginalis* trophozoites. Parasite viability was determined by CellTiter-Glo^®^, microscopy, and rescue–transformation assays, and selectivity indices (SI) were calculated. Amphotericin B and metronidazole served as reference drugs.

**Results:**

Both extracts exhibited low cytotoxicity in THP-1 macrophages (*A. vulgaris* CC₅₀ = 465.2 µg/mL; *B. vulgaris* = 357.7 µg/mL). Against *L. major*, *B. vulgaris* showed greater potency (IC₅₀ = 76.8 µg/mL; SI = 4.7 for amastigotes) than *A. vulgaris* (IC₅₀ = 179.7 µg/mL; SI = 2.6). Both extracts reduced intracellular parasite burden in a dose-dependent manner, achieving complete clearance at non-cytotoxic concentrations (≥ 300 µg/mL). In *T. vaginalis*, the extracts induced concentration-dependent inhibition, with IC₅₀ values of 68.9 µg/mL (*B. vulgaris*, SI = 5.2) and 104.4 µg/mL (*A. vulgaris*, SI = 4.5).

**Conclusion:**

Both extracts exhibited selective, dose-dependent antiprotozoal activity, with *B. vulgaris* showing superior efficacy, particularly against intracellular *L. major* and *T. vaginalis*. These results highlight their potential as natural antiprotozoal sources, warranting further studies on active constituents, mechanisms, and in vivo efficacy.

**Supplementary Information:**

The online version contains supplementary material available at 10.1007/s11686-026-01224-1.

## Introduction

Neglected protozoan diseases, particularly those caused by *Trichomonas vaginalis* and *Leishmania* species, continue to impose a substantial global health burden. These infections disproportionately affect socioeconomically disadvantaged populations, especially in low- and middle-income countries where limited access to healthcare exacerbates their impact [1]. *T. vaginalis* and *Leishmania* spp., unicellular eukaryotic parasites within the supergroup Excavata, collectively account for hundreds of millions of infections annually, yet remain underrepresented in drug discovery pipelines [2, 3].

Leishmaniasis manifests in cutaneous, visceral, and mucocutaneous forms, with cutaneous leishmaniasis (CL) being the most prevalent. The World Health Organization (WHO) estimates that more than 15 million individuals are infected with Leishmania parasites worldwide, with ~ 2 million new cases each year and over 350 million people at risk [4]. Pentavalent antimonials such as sodium stibogluconate and meglumine antimoniate remain first-line treatments, though their use is limited by prolonged administration, systemic toxicities, and 10–15% therapeutic failure rates [5]. Alternative therapies—including amphotericin B, miltefosine, and paromomycin—are available, but issues of cost, parenteral administration, and emerging drug resistance restrict their widespread adoption.

Trichomoniasis, caused by *T. vaginalis*, is the most common non-viral sexually transmitted infection, with an estimated 156 million new cases annually [6]. The disease is associated with significant reproductive morbidity, including preterm delivery and low birth weight, as well as increased susceptibility to HIV and cervical neoplasia [7]. Metronidazole remains the standard therapy; however, concerns regarding gastrointestinal intolerance, genotoxicity, reproductive toxicity, poor treatment adherence, and increasing resistance among *T. vaginalis* strains highlight the urgent need for alternative therapeutic options [8, 9]. These challenges mirror the broader phenomenon of emerging drug resistance across protozoan parasites, which threatens the long-term effectiveness of current chemotherapeutic strategies.

Against this backdrop, natural products—particularly medicinal plants—have re-emerged as valuable reservoirs for antiparasitic drug discovery. Plant-derived phytochemicals often exhibit favorable safety profiles and diverse pharmacological activities, reflecting centuries of use in traditional medicine [10]. Among these, species of the *Artemisia* genus (Asteraceae) are renowned for their antiparasitic potential, most notably Artemisia annua as the source of artemisinin [11]. *Artemisia vulgaris*, a closely related species, demonstrates antimicrobial, anticancer, and anti-inflammatory activities, though its antiprotozoal properties remain underexplored. Similarly, *Berberis vulgaris* (Berberidaceae), rich in the isoquinoline alkaloid berberine, has been widely used in ethnomedicine for infectious, cardiovascular, and gastrointestinal disorders [12]. While berberine and related alkaloids display antiparasitic effects, systematic evaluation of *B. vulgaris* extracts against protozoan pathogens is limited.

Given the high global prevalence of leishmaniasis and trichomoniasis and the shortcomings of current therapies, there is a compelling need to identify plant-based alternatives. To our knowledge, no study has comprehensively evaluated the antiprotozoal activities of *A. vulgaris* and *B. vulgaris* against *L. major* and *T. vaginalis* in parallel. Therefore, this study aimed to assess the in vitro efficacy of extracts from these two medicinal plants against *L. major* promastigotes and intracellular amastigotes, as well as *T. vaginalis* trophozoites, while simultaneously examining host cell cytotoxicity. Ultimately, this work provides preliminary yet essential evidence for the potential of *A. vulgaris* and *B. vulgaris* as accessible and effective candidates in the treatment of neglected protozoan diseases.

## Material and Methods

### Ethical Statement

The study was approved by the Clinical Research Ethics Committee of Manisa Celal Bayar University, Faculty of Medicine (Date: 25 March 2021; Decision No: E.49155). The experiments involved established cell lines and laboratory-maintained protozoan isolates; no human participants were directly recruited, and informed consent was therefore not applicable.

### Plant Materials

**Fig. 1:**
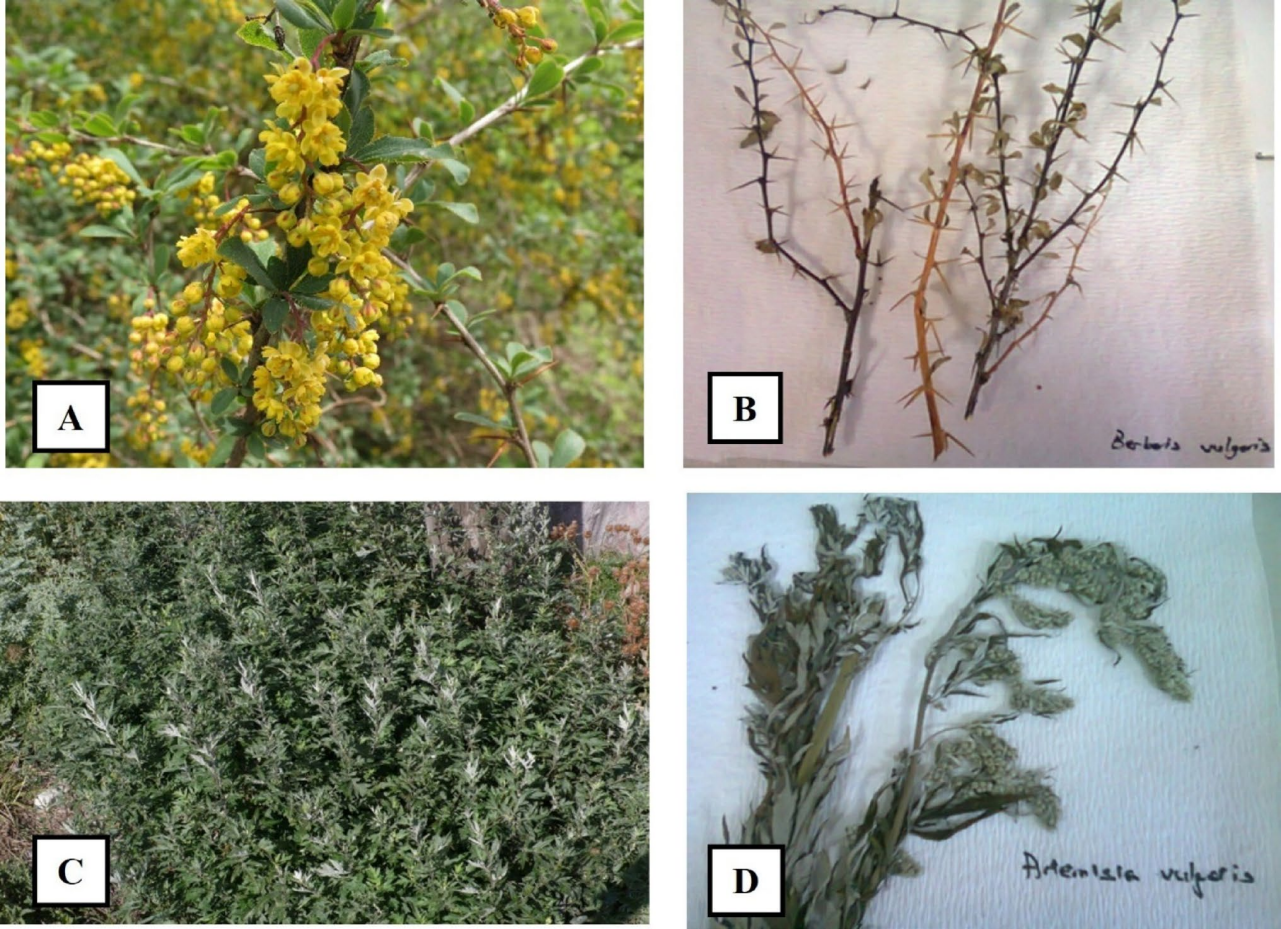
Morphological characteristics and herbarium specimens of the studied medicinal plants. **A** *Berberis vulgaris* (Berberidaceae) in natural habitat, showing racemose yellow flowers. **B** Herbarium specimen of *B. vulgaris* aerial parts collected from Spil Mountain, Manisa, Türkiye. **C** *Artemisia vulgaris* (Asteraceae) in natural habitat, displaying its characteristic bushy growth. **D** Herbarium specimen of *A. vulgaris* aerial parts collected from Ardahan, Türkiye

*Artemisia vulgaris* (Asteraceae) and *Berberis vulgaris* (Berberidaceae) aerial parts (above-ground portions including stems and leaves) were collected from natural populations in Türkiye. Samples of *A. vulgaris* were obtained from Ardahan (Central dis trict, 41°06′42″N, 42°42′47″E; 1820 m altitude), while *B. vulgaris* specimens were collected from Manisa, Spil Mountain (138°33′49″N, 27°26′38″E; 1310 m altitude). Taxonomic identification was performed and confirmed by Dr. Cenk Durmuskahya (Izmir Kâtip Celebi University, Faculty of Forestry, Department of Forest Engineering, İzmir, Türkiye) (Fig. 1)**.** The collected plant materials were shade-dried at room temperature, ground into fine powder, and extracted with ethanol by maceration for 72 h at room temperature with intermittent stirring. Ultrasonic-assisted extraction was additionally employed to enhance extraction efficiency. The filtrates were concentrated under reduced pressure using a rotary evaporator and lyophilized to dryness. Extraction yields (%) were calculated based on the dry weight of the original plant material. Lyophilized extracts were stored at − 80 °C until further use to ensure stability. For biological assays, dried extracts were reconstituted in dimethyl sulfoxide (DMSO) (Sigma Alrich^®^, ABD), ensuring a maximum final solvent concentration of ≤ 0.5% (v/v) in experimental wells. Equivalent concentrations of DMSO were included as vehicle controls in all assays [13].

### Culture Media Preparation

*Trichomonas vaginalis* trophozoites were cultured in TYM (trypticase–yeast extract–maltose) medium; prepared according to Diamond’s formulation, supplemented with 10% (v/v) heat-inactivated fetal calf serum (FCS; Gibco^®^, USA). Medium pH was adjusted to 6.0, sterilized by autoclaving (121 °C, 15 min), and stored at 4 °C until use.

NNN (Novy-MacNeal-Nicolle) biphasic medium was first prepared by autoclaving agar, peptone, and NaCl in distilled water (121 °C, 20 min). After cooling, defibrinated rabbit blood and antibiotics (penicillin/streptomycin (Genemarkbio^®^, China) and gentamicin (Thermo Fisher^®^, USA) were added. Three-millilitre portions were poured into sterile slant tubes, solidified at a 10° angle, and stored at 4 °C. Before use, each slant was overlaid with 1 mL RPMI-1640 (Gibco^®^, USA) containing 10% FCS. *Leishmania major* promastigotes were then cultured in this biphasic system using RPMI-1640 supplemented with 10% FCS, 1% penicillin/streptomycin (Genemarkbio^®^, China), and 1% gentamicin, and freshly prepared cultures were used for experiments.

### THP-1 Cell Culture and Differentiation

**Fig. 2:**
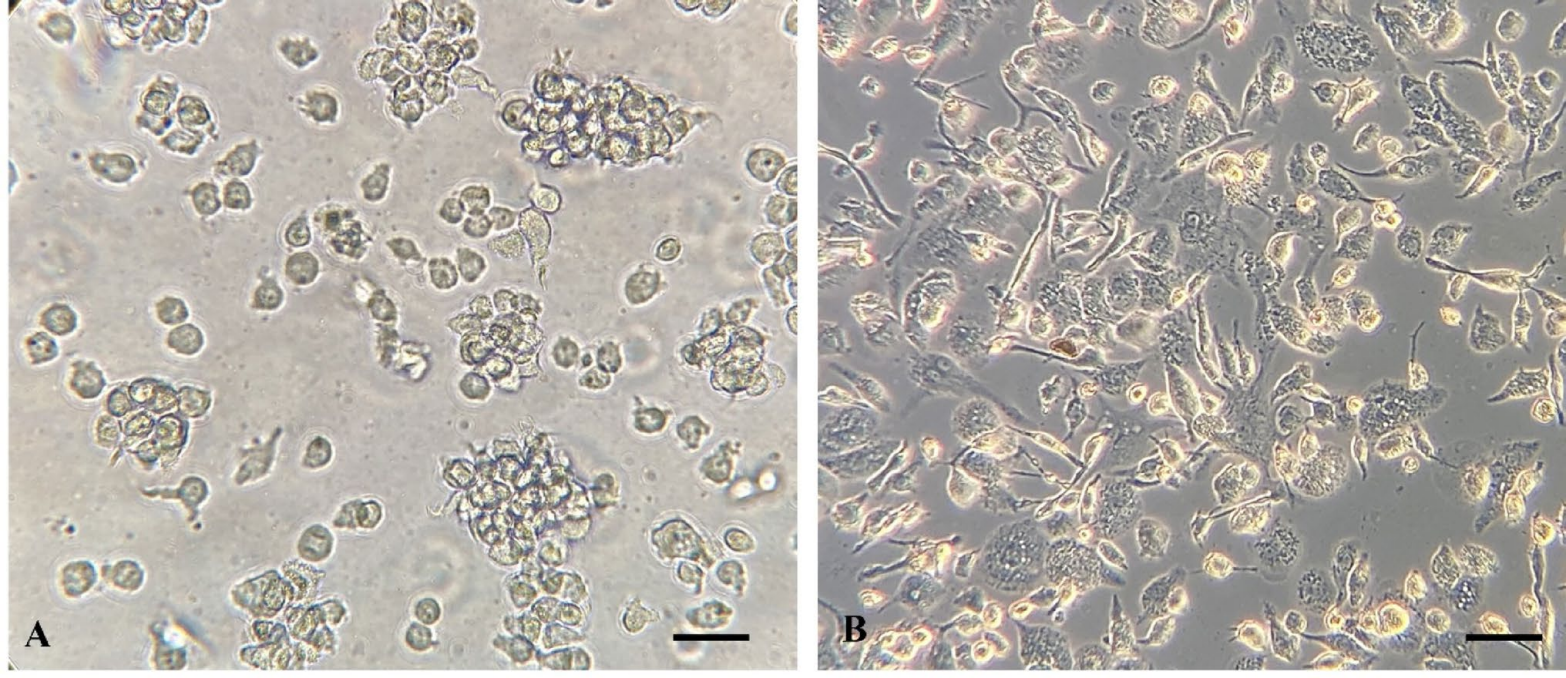
Differentiation of THP-1 monocytes into macrophage-like cells. **A** Undifferentiated THP-1 monocytes with round, suspended morphology. **B** THP-1 cells after stimulation with PMA (100 ng/mL, 24 h), showing adherence and elongated macrophage-like morphology. Phase-contrast microscopy, 400 × ; scale bar = 10 µm

The human monocytic cell line THP-1 (ATCC^®^ TIB-202™, obtained from the Parasite Bank, Manisa Celal Bayar University) was cultured in RPMI-1640 medium supplemented with 10% FCS, 1% penicillin/streptomycin, and 1% L-glutamine (Thermo Fisher Scientific^®^, ABD) at 37 °C in a humidified 5% CO₂ atmosphere (Thermo Fisher^®^, USA). Cell viability was confirmed by trypan blue (Sigma Alrich^®^, USA) exclusion, and only cultures with ≥ 95% viable cells were used. For differentiation into macrophage-like cells, THP-1 monocytes (1 × 10⁶ cells/mL) were treated with 100 ng/mL phorbol 12-myristate 13-acetate (PMA; Sigma-Aldrich, USA) for 24 h, followed by 72 h incubation in fresh medium without PMA. Differentiation was verified by adherence and characteristic morphological changes under phase-contrast microscopy (Zeiss Primovert, Germany) (Fig. 2).

### Parasite Culture and Maintenance

**Fig. 3:**
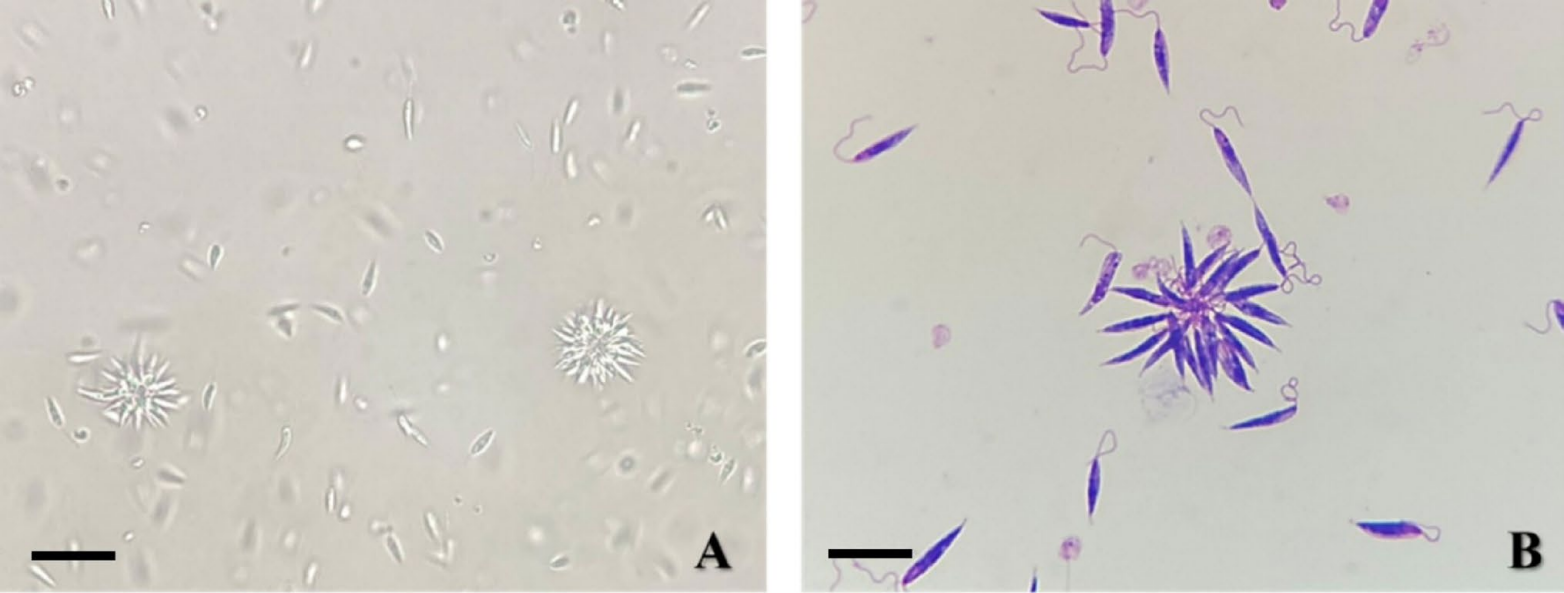
Morphology of *Leishmania major* promastigotes. **A** Unstained promastigotes showing motile cells and characteristic rosette formation under phase-contrast microscopy (400 ×). **B** Giemsa-stained promastigotes displaying elongated bodies with visible flagella and rosette structures (1000 × , oil immersion). Scale bar = 10 µm

*Leishmania major* (MHOM/1973/5ASKH) and *Trichomonas vaginalis* (ATCC^®^ 50143™) isolates, cryopreserved in the Parasite Bank (Department of Medical Parasitology, Manisa Celal Bayar University), were used. Parasites were thawed at 37 °C and transferred into pre-warmed medium under aseptic conditions. *L. major* promastigotes were first inoculated into NNN biphasic medium at 26 °C and subsequently subcultured in RPMI-1640 medium supplemented with 10% FCS, 1% penicillin/streptomycin, and 1% L-glutamine. Cultures were maintained at 26 °C and used in experiments during the logarithmic growth phase (Fig. 3).

**Fig. 4:**
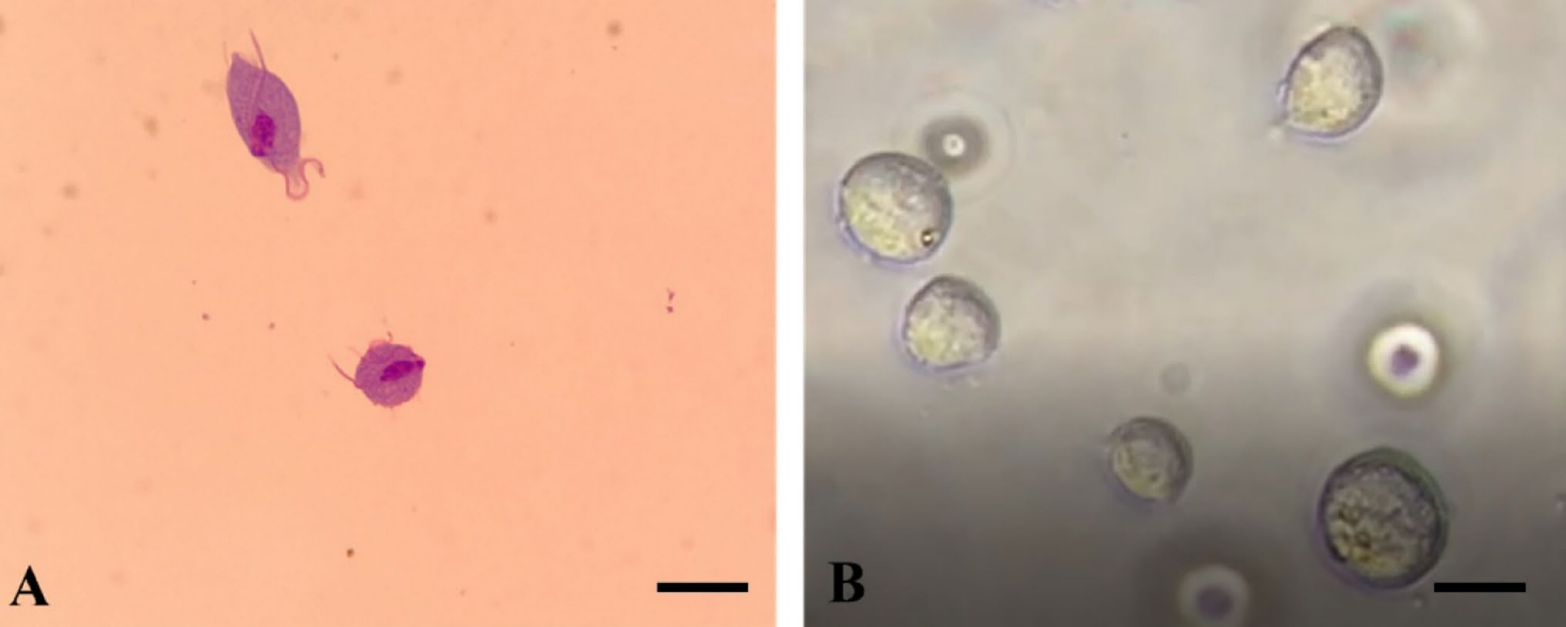
Morphology of *Trichomonas vaginalis* trophozoites. **A** Giemsa-stained trophozoites showing oval morphology with a central nucleus and visible flagella under light microscopy (1000 × , oil immersion). **B** Unstained trophozoites observed as rounded forms in suspension under phase-contrast microscopy (400 ×). Scale bar = 10 µm

*T. vaginalis* trophozoites were cultured in TYM medium supplemented with 10% FCS (pH 6.0) at 37 °C and subcultured every 48 h. Logarithmic-phase trophozoites exhibiting motility and normal morphology were selected for assays (Fig. 4).

### Evaluation of Cytotoxic Effects on THP-1 Macrophages

Cytotoxicity of *Artemisia vulgaris* and *Berberis vulgaris* extracts was evaluated in differentiated THP-1 macrophages using the CellTiter-Glo^®^ Luminescent Cell Viability Assay (Promega, USA), following the manufacturer’s instructions. Cells (5 × 10^5^/mL) were seeded in white opaque, flat-bottom 96-well microplates (Thermo Fisher^®^, Denmark) and exposed to graded extract concentrations (1000–15 µg/mL) for 24 h and 48 h at 37 °C in 5% CO₂. Amphotericin B (AmB; Sigma-Aldrich, USA) and metronidazole (Sigma-Aldrich, USA) were included as reference drugs, while vehicle controls contained 0.5% DMSO. Luminescence was recorded using a microplate reader, and cell viability was expressed relative to untreated controls. The 50% cytotoxic concentration (CC₅₀) was calculated from nonlinear regression of dose–response curves (GraphPad Prism v9.1 Software, San Diego, CA, USA). All assays were performed in triplicate and repeated at least three times.

### Antileishmanial Activity Against *Leishmania major* Promastigotes

Log-phase *L. major* promastigotes (1 × 10⁷/mL) were incubated with serial dilutions of *Artemisia vulgaris* and *Berberis vulgaris* extracts (1000–15 µg/mL) in white opaque, flat-bottom 96-well microplates (Thermo Fisher^®^, Denmark). Parasite viability was assessed after 24 h and 48 h at 26 ± 1 °C using the CellTiter-Glo^®^ Luminescent Cell Viability Assay. Amphotericin B was used as a reference drug, while untreated cultures and medium-only wells served as growth and blank controls. The 50% inhibitory concentration (IC₅₀) was determined by nonlinear regression of dose–response curves (GraphPad Prism v9.1 Software, San Diego, CA, USA). All experiments were performed in triplicate and repeated at least three times.

### Antileishmanial Activity Against *Leishmania major* Intracellular Amastigotes

Differentiated THP-1 macrophages (5 × 10^5^/well) were infected with log-phase *L. major* promastigotes at a macrophage-to-parasite ratio of 1:10 for 24 h at 37 °C in 5% CO₂. Non-internalized parasites were removed, and infected cells were treated with *A. vulgaris* and *B. vulgaris* extracts (300–50 µg/mL) or AmB (1–0.03 µM) as reference drug. After 24 h and 48 h, cells were methanol-fixed, Giemsa-stained, and the intracellular parasite burden was determined microscopically by counting 100 macrophages per coverslip. The percentage of infected cells and mean amastigotes per macrophage were recorded. The 50% inhibitory concentration (IC₅₀) was calculated by nonlinear regression relative to untreated controls (GraphPad Prism v9.1 Software, San Diego, CA, USA). All assays were performed in triplicate and repeated at least three times.

### Quantitative Cell Viability and Parasite Rescue and Transformation Assay (PRTA)

The assay was adapted from Jain et al. [14] with minor modifications. Differentiated THP-1 macrophages were infected with log-phase *L. major* promastigotes at a multiplicity of infection (MOI) of 1:10 for 48 h at 37 °C in 5% CO₂. After removal of non-internalized parasites, infected cells were treated with serial concentrations of *A. vulgaris* and *B. vulgaris* extracts (300–50 µg/mL) or AmB (1–0.03 µM) as a reference drug for 48 h. Following treatment, macrophages were lysed by adding Schneider’s Insect Medium (SIM) containing 0.05% SDS (Sodium Dodecyl Sulfate), and the released amastigotes were allowed to transform into promastigotes by incubation in SIM supplemented with 10% FCS at 26 ± 1 °C for 72 h. Parasite viability was then quantified using the CellTiter-Glo^®^ Luminescent Cell Viability Assay. The half-maximal inhibitory concentration (IC₅₀) was calculated by nonlinear regression analysis of dose–response curves (GraphPad Prism v9.1 Software, San Diego, CA, USA). All experiments were performed in triplicate and repeated independently.

### Antitrichomonal Activity Against *Trichomonas vaginalis* Trophozoites

Log-phase *T. vaginalis* trophozoites were exposed to serial concentrations of *A. vulgaris* and *B. vulgaris* extracts (1000–15 µg/mL) or metronidazole (500–15 µg/mL) as a reference drug in TYM medium at 37 °C. Drug-free cultures served as negative controls. Parasite viability was assessed by trypan blue exclusion and microscopic evaluation of motility, and growth inhibition was expressed relative to untreated controls. In parallel, viability was quantified using the CellTiter-Glo^®^ Luminescent Cell Viability Assay. The 50% inhibitory concentration (IC₅₀) was determined from nonlinear regression of dose–response curves (GraphPad Prism v9.1 Software, San Diego, CA, USA). All experiments were performed in triplicate and repeated independently.

### Selectivity Index (SI)

The selectivity index (SI) was calculated as the ratio of the 50% cytotoxic concentration (CC₅₀) in THP-1 host cells to the 50% inhibitory concentration (IC₅₀) against the parasite (SI = CC₅₀/IC₅₀) [15, 16]. SI values were determined for *A. vulgaris* and *B. vulgaris* extracts against *T. vaginalis* trophozoites and both promastigote and intracellular amastigote stages of *L. major*.

### Statistical Analysis

All experiments were performed in triplicate and repeated at least three times. Data were expressed as mean ± standard deviation (SD). Dose–response curves for the determination of CC₅₀ (THP-1 macrophages), IC₅₀ (promastigotes, intracellular amastigotes, and trophozoites), and SI values were generated by nonlinear regression analysis using GraphPad Prism v9.1.(GraphPad Software, San Diego, CA, USA). Statistical comparisons between treatment groups and controls were performed using one-way analysis of variance (ANOVA) followed by Tukey’s post hoc test. For non-parametric data, the Kruskal–Wallis test was applied with Dunn’s multiple comparison correction. Differences were considered statistically significant at *p* < 0.05.

## Results

### Cytotoxicity Evaluation of *A. vulgaris *and *B. vulgaris* in THP-1 Macrophages

**Fig. 5:**
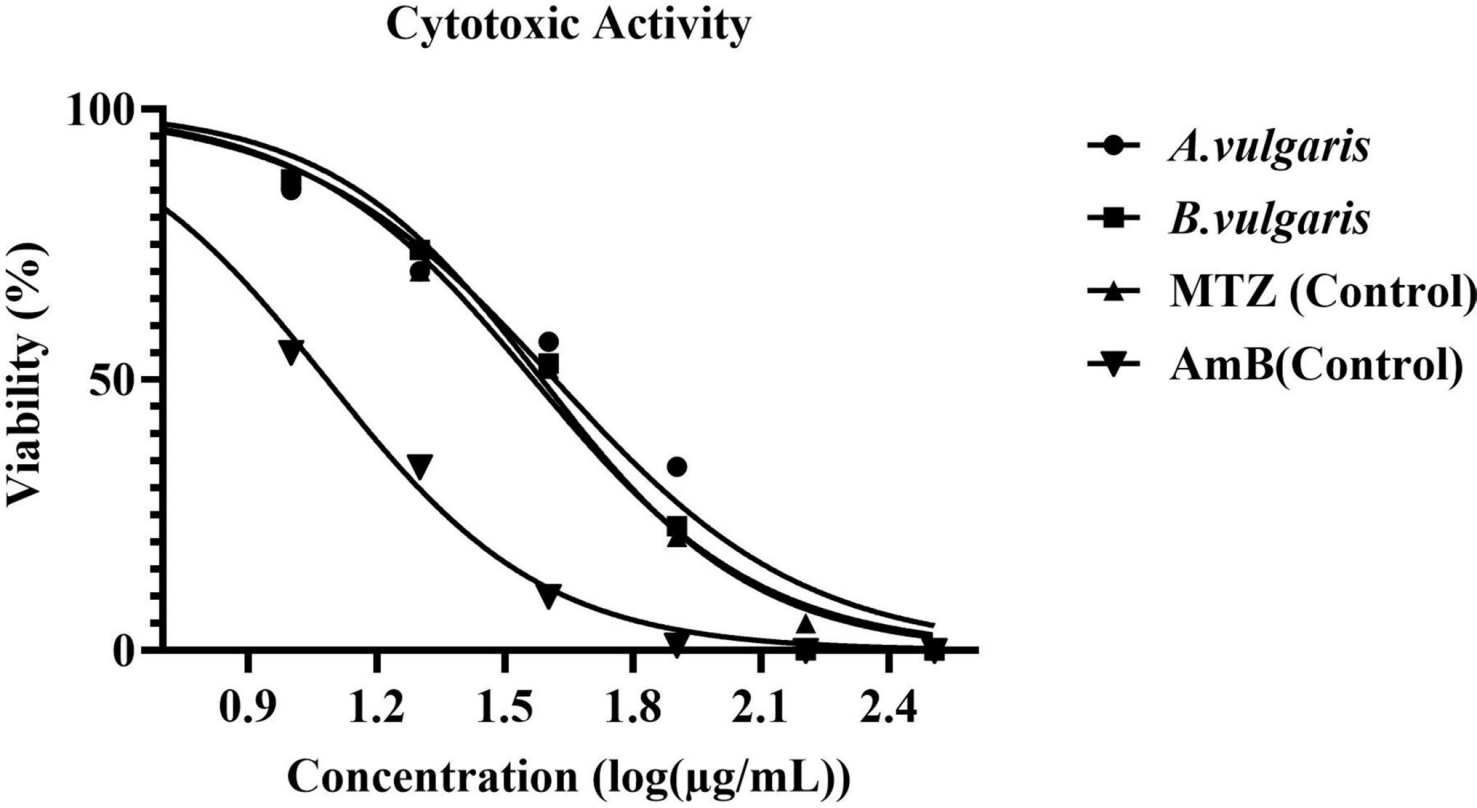
Cytotoxic activity of *Artemisia vulgaris* and *Berberis vulgaris* plant extracts against THP-1-derived macrophages

The cytotoxic potential of *A. vulgaris* and *B. vulgaris* extracts on THP-1 macrophages was assessed using the CellTiter-Glo luminescent assay, and data were analyzed statistically. Both extracts demonstrated low cytotoxicity, with CC₅₀ values of 465.2 ± 38.7 µg/mL for *A. vulgaris* and 357.7 ± 30.2 µg/mL for *B. vulgaris*. Amphotericin B (AmB) and metronidazole (MTZ) were used as reference drugs, with CC₅₀ values of 15.0 ± 0.6 µM (13.9 ± 0.6 µg/mL) and 255.6 ± 12 µM (43.75 ± 2.05 µg/mL), respectively, in THP-1 macrophages (Fig. 5). These results indicate that neither extract exhibited significant cytotoxic effects within the tested concentration range.

### Antileishmanial Activity of *A. vulgaris *and *B. vulgaris* Against *L. major* Promastigotes

**Fig. 6:**
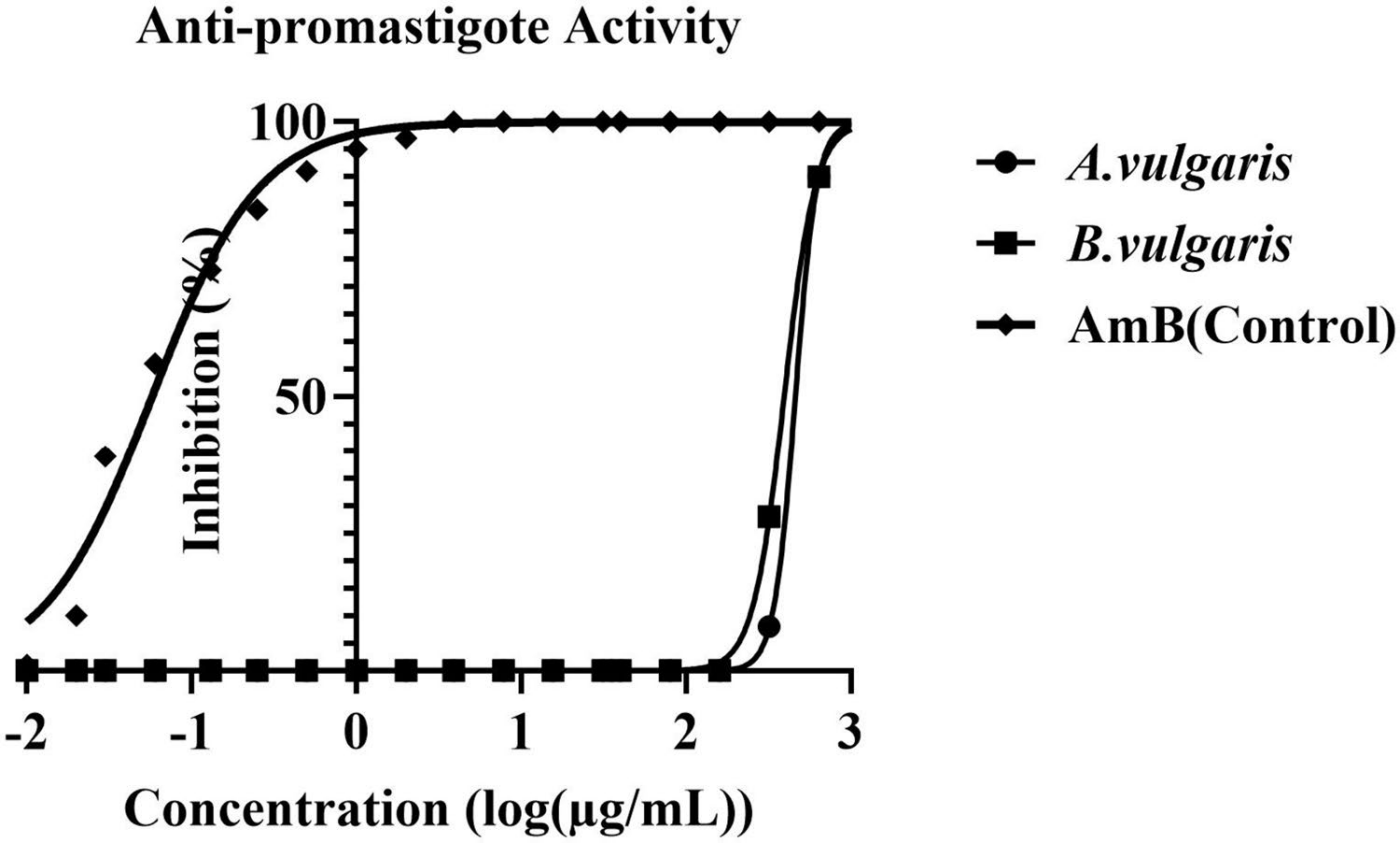
In vitro antileishmanial activity (IC_50_) of *Artemisia vulgaris* and *Berberis vulgaris* plant extracts against *Leishmania major* promastigotes

The antileishmanial potential of *A. vulgaris* and *B. vulgaris* extracts was assessed against *L. major* promastigotes using CellTiter-Glo luminescent assays following 48 h of incubation. Amphotericin B (AmB) was used as the reference control, exhibiting an IC_50_ value of 0.05 µM (0.046 ± 0.0019 µg/mL). The extracts displayed moderate inhibitory activity, with IC_50_ values of 460.9 ± 38.5 µg/mL for *A. vulgaris* and 352.0 ± 28.7 µg/mL for *B. vulgaris* (Fig. 6).

### Antileishmanial Activity of *A. vulgaris *and *B. vulgaris* Extracts Against *L. major* Intracellular Amastigotes

Microscopic examination of Giemsa-stained control slides revealed an intracellular parasite burden of 3.43 ± 0.12 amastigotes per macrophage, with 39% of macrophages infected. Treatment with *B. vulgaris* extracts at concentrations of 50, 75, and 100 µg/mL resulted in a dose-dependent decrease in amastigote load, reducing the parasite burden to 2.63 ± 0.24, 2.20 ± 0.18, and 1.76 ± 0.21 amastigotes per macrophage, respectively, with corresponding infection rates of 30%, 25%, and 20%. At higher concentrations (150–300 µg/mL), no intracellular amastigotes were detected, and macrophage infection rates dropped to 0%.

**Fig. 7:**
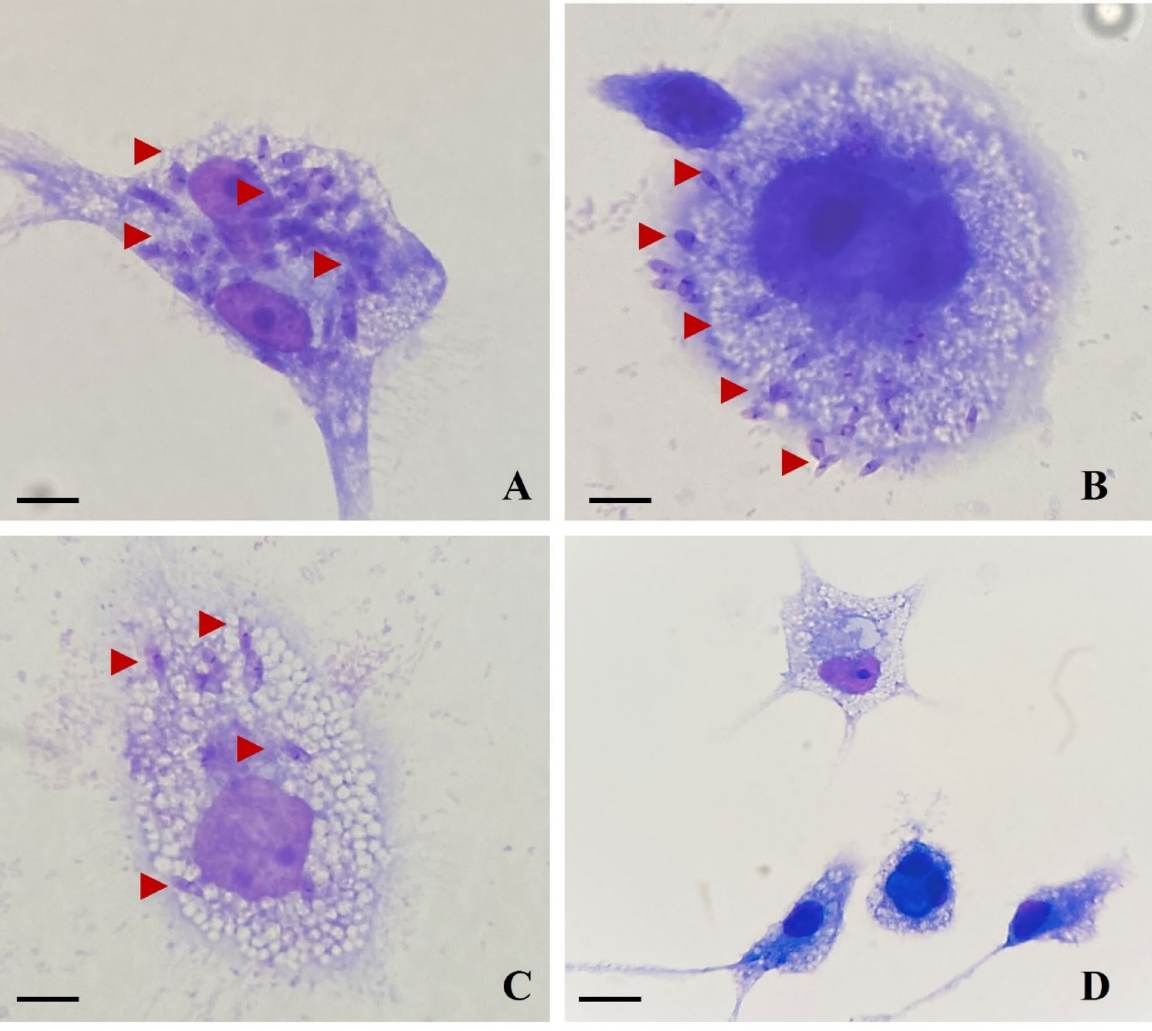
Giemsa-stained THP-1 macrophages infected with *Leishmania major* and treated with plant extracts. **A** Untreated control macrophage showing heavy intracellular infection with numerous amastigotes (red arrows). **B** Macrophage treated with *Artemisia vulgaris* extract exhibiting reduced parasite burden. **C** Macrophage treated with *Berberis vulgaris* extract displaying markedly decreased intracellular amastigotes. **D** Uninfected THP-1 macrophages demonstrating normal cell morphology and absence of intracellular parasites. Images captured at 1000 × magnification; scale bar = 10 μm

Similarly, exposure to *A. vulgaris* extracts produced a concentration-dependent inhibition of amastigote proliferation. At 50, 75, 100, 150, and 200 µg/mL, the mean parasite load decreased progressively to 2.46 ± 0.22, 2.37 ± 0.23, 2.11 ± 0.20, 1.85 ± 0.19, and 1.58 ± 0.16 amastigotes per macrophage, with infection rates of 28%, 27%, 24%, 21%, and 18%, respectively. Complete clearance of intracellular amastigotes was observed at 300 µg/mL, where the infection rate reached 0% (Table 1) (Fig. 7).

**Table 1:** Effect of different concentrations of *Berberis vulgaris* and *Artemisia vulgaris* extracts on of *Leishmania major* intracellular amastigotes in comparison to the control group

Concentrations (µg/mL)	*Artemisia vulgaris*			*Berberis vulgaris*		
	Amastigote rate (per macrophage)		% Infected macrophage	Amastigote rate (per macrophage)		% Infected macrophage
	M	SD		M	SD	
300	0*	0*	0*	0*	0*	0*
200	1.58*	0.16*	18*	0*	0*	0*
150	1.85*	0.19*	21*	0*	0*	0*
100	2.11*	0.20*	24*	1.76*	0.21*	20*
75	2.37*	0.23*	27*	2.20*	0.18*	25*
50	2.46*	0.22*	28*	2.63*	0.24*	30*
Control	3.43	0.12	39	3.43	0.12	39

*Data are expressed as mean (M) ± standard deviation (SD) of three independent experiments performed in triplicate. The intracellular amastigote burden was determined by microscopic examination of Giemsa-stained preparations, with a minimum of 100 macrophages counted per coverslip. Statistical analysis was performed using one-way ANOVA followed by Tukey’s post hoc test. *p* < 0.05 indicates statistically significant differences compared with the untreated control group

### Parasite Rescue and Transformation Assay (Transformed Promastigotes)

**Fig. 8:**
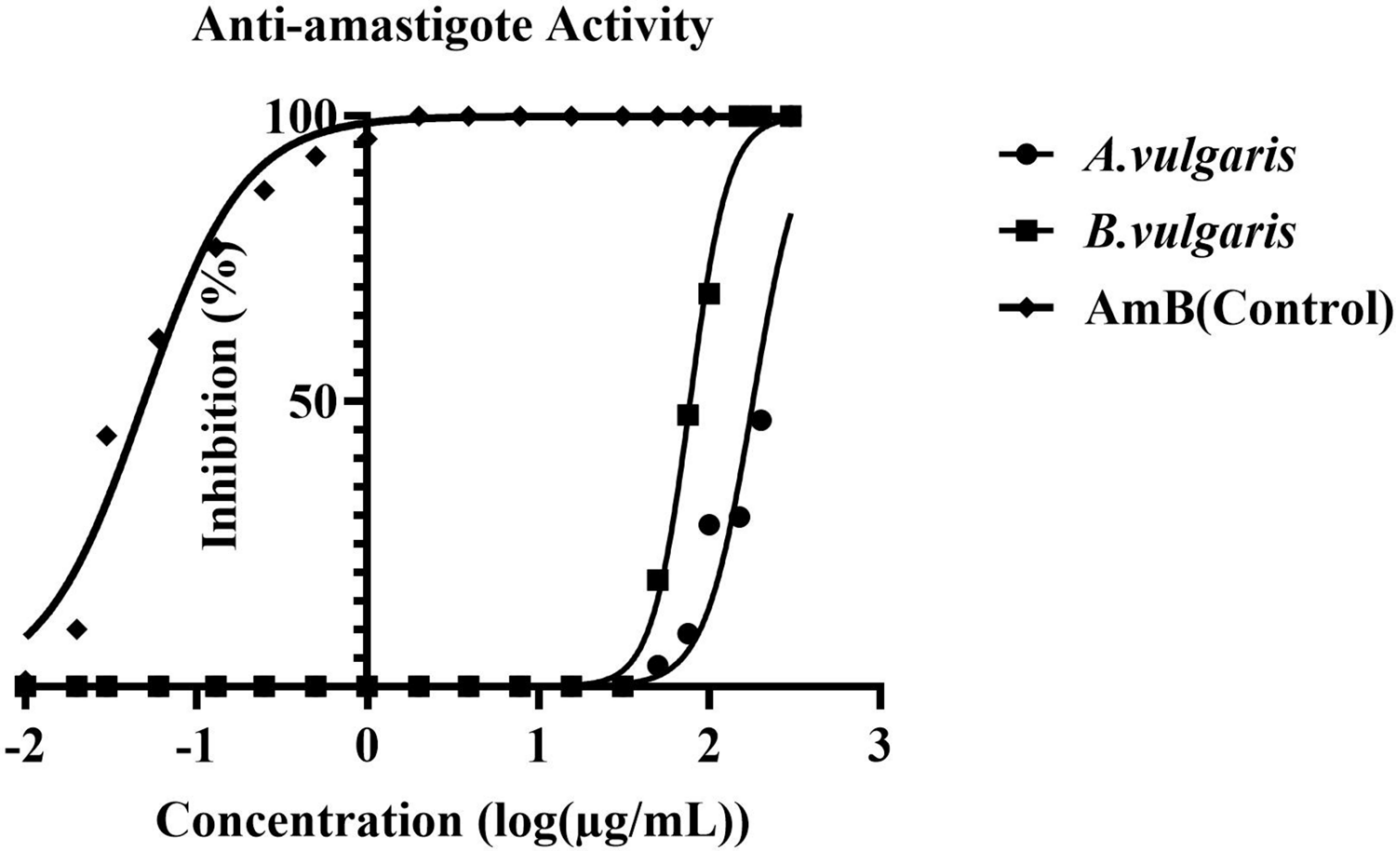
Ex vivo antileishmanial activity (IC_50_) of *Artemisia vulgaris* and *Berberis vulgaris* plant extracts against intracellular *Leishmania major* amastigotes

To further assess the ex vivo efficacy of the plant extracts, a parasite rescue and transformation assay was conducted to quantify the viability of intracellular amastigotes. The IC₅₀ value of *B. vulgaris* was determined to be 76.8 ± 6.3 µg/mL, whereas *A. vulgaris* exhibited an IC₅₀ of 179.7 ± 15.2 µg/mL. In comparison, the reference drug AmB demonstrated markedly higher potency, with an IC₅₀ value of 0.04 ± 0.001 µM (0.037 ± 0.0009 µg/mL). Quantitative analysis confirmed that both extracts exerted significant, dose-dependent inhibitory effects compared with the untreated control group (p < 0.05). These findings indicate that *B. vulgaris* and *A. vulgaris* extracts effectively suppress the intracellular proliferation of *L. major* amastigotes, albeit with lower potency than AmB (Fig. 8).

### Antitrichomonal Activity of *A. vulgaris *and *B. vulgaris* Against *T. vaginalis* Trophozoites

**Fig. 9:**
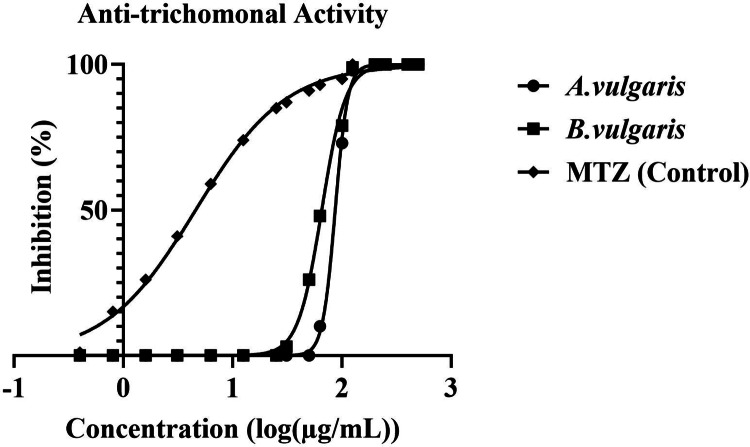
In vitro antitrichomonal activity IC_50_ of *Artemisia vulgaris* and *Berberis vulgaris* plant extracts against *Trichomonas vaginalis* trophozoites

The antitrichomonal effects of *A. vulgaris* and *B. vulgaris* extracts on *T. vaginalis* trophozoites were assessed after 48 h of incubation by combining microscopic evaluation, hemocytometric counting with trypan blue exclusion, and the CellTiter-Glo^®^ luminescent viability assay. Drug-free cultures served as negative controls, whereas metronidazole (MTZ; 500–15 µg/mL) was used as a reference standard. Microscopic observations revealed a concentration-dependent reduction in trophozoite motility and evidence of cell lysis (Table 2). At higher concentrations (200–250 µg/mL), both extracts induced complete parasite disintegration, with no motile trophozoites detected. At 125 µg/mL, partial inhibition was evident: motility was markedly reduced, and intracellular degeneration was observed. Lower concentrations (50–100 µg/mL) caused moderate suppression of motility, while trophozoite morphology appeared abnormal compared with untreated controls. Quantitative analysis further substantiated these findings (Table 3). Hemocytometric counts demonstrated that both extracts reduced trophozoite numbers significantly relative to the control (p < 0.05). Complete growth inhibition (100%) was achieved at 200–250 µg/mL for both *A. vulgaris* and *B. vulgaris*. At 125 µg/mL, trophozoite counts decreased to 0.4–0.6 ± 0.1 × 10^4^ cells (~ 85–90% inhibition). Concentrations of 100, 62.5, and 50 µg/mL yielded parasite counts of approximately 1.0–1.3 ± 0.2 (~ 70% inhibition), 1.5–1.9 ± 0.3 (~ 55–63% inhibition), and 1.8–2.1 ± 0.3 × 10^4^ (~ 48–56% inhibition), respectively, compared with 4.0–4.2 ± 0.3 × 10^4^ trophozoites in untreated control cultures. The CellTiter-Glo assay corroborated these results, yielding IC_50_ values of 104.4 ± 9.2 µg/mL for *A. vulgaris* and 68.9 ± 6.1 µg/mL for *B. vulgaris*. As shown in the dose–response curve (Fig. 9), both extracts exhibited significant inhibitory activity in a concentration-dependent manner, with *B. vulgaris* demonstrating greater potency, consistent with its lower IC₅₀ value. MTZ displayed superior efficacy, achieving complete inhibition at lower concentrations, but the extracts nonetheless produced statistically significant suppression of parasite viability compared with the untreated control group (*p* < 0.05).

**Table 2:** Effect of *Artemisia vulgaris* and *Berberis vulgaris* extracts on the motility and lysis of *Trichomonas vaginalis* trophozoites across various concentrations and incubation periods, as observed under a light microscope

Concentration (µg/mL)	*	*Artemisia vulgaris*		*Berberis vulgaris*	
		24 h	48 h	24 h	48 h
250	Movement	−	−	−	−
	Lysis	+	+	+	+
200	Movement	−	−	−	−
	Lysis	+	+	+	+
125	Movement	±	−	±	−
	Lysis	−	+	−	+
100	Movement	+	±	±	±
	Lysis	−	−	−	−
62.5	Movement	+	+	+	±
	Lysis	−	−	−	−
50	Movement	+	+	+	+
	Lysis	−	−	−	−

*“Movement” indicates observed trophozoite motility under light microscopy; “Lysis” indicates cell disintegration. Symbols: “+” = presence; “±” = reduced or weak activity; “−” = absence

**Table 3:** Quantitative assessment of mean trophozoite count ± SD (× 10^4^) and percentage growth inhibition of *Trichomonas vaginalis* following exposure to various concentrations of *Artemisia vulgaris* and *Berberis vulgaris* extracts relative to the untreated control

Concentrations (µg/mL)	*Artemisia vulgaris* 24 h (M ± SD)/%GI	*Artemisia vulgaris* 48 h (M ± SD)/%GI	*Berberis vulgaris* 24 h (M ± SD)/%GI	*Berberis vulgaris* 48 h (M ± SD)/%GI
250	0.0 ± 0.0*/100%	0.0 ± 0.0*/100%	0.0 ± 0.0*/100%	0.0 ± 0.0*/100%
200	0.0 ± 0.0*/100%	0.0 ± 0.0*/100%	0.0 ± 0.0*/100%	0.0 ± 0.0*/100%
125	0.6 ± 0.15*/84%	0.4 ± 0.12*/90%	0.5 ± 0.14*/86%	0.3 ± 0.10*/92%
100	1.3 ± 0.20*/68%	1.1 ± 0.18*/73%	1.0 ± 0.22*/75%	0.9 ± 0.16*/78%
62.5	1.9 ± 0.25*/53%	1.6 ± 0.21*/60%	1.7 ± 0.24*/58%	1.5 ± 0.20*/63%
50	1.9 ± 0.25*/53%	1.9 ± 0.25*/55%	2.0 ± 0.27*/51%	1.8 ± 0.22*/56%
Control (NTC)	4.1 ± 0.32/0%	4.2 ± 0.30/0%	4.0 ± 0.31/0%	4.1 ± 0.33/0%

*Values are expressed as mean (M) ± standard deviation (SD) of three independent experiments. Statistical analysis was performed using one-way ANOVA followed by Tukey’s post hoc test. *p* < 0.05 indicates statistically significant differences compared with the non-treated control (NTC) at the same incubation time. %GI: percentage growth inhibition

### Selectivity Index (SI)

The selectivity indices (SI) of *A. vulgaris* and *B. vulgaris* extracts were calculated using their IC₅₀ values for *Leishmania major* promastigotes (IC₅₀) and amastigotes (IC₅₀), as well as the CC₅₀ values obtained in THP-1 macrophages. For *A. vulgaris*, the SI was 1.0 for promastigotes and 2.6 for amastigotes, indicating limited selectivity towards the intracellular stage. In contrast, *B. vulgaris* displayed SI values of 1.01 for promastigotes and 4.7 for amastigotes, reflecting a comparatively higher selectivity for the clinically relevant amastigote stage (Table 4 ). Similarly, SI values were determined for *T. vaginalis* trophozoites based on IC₅₀₍troph₎ and CC₅₀ values. The SI of *A. vulgaris* was calculated as 4.5, while *B. vulgaris* exhibited an SI of 5.2, suggesting that both extracts exert selective antitrichomonal activity, with *B. vulgaris* showing slightly greater safety margins relative to host cells (Tables 5).

**Table 4:** Selectivity indices (SI) of *Artemisia vulgaris* and *Berberis vulgaris* extracts against *Leishmania major* promastigotes and amastigotes compared with amphotericin B

Extracts	IC_50_ (µg/mL)		CC_50_ (µg/mL)	SI (CC₅₀/IC₅₀)	
	Promastigote	Amastigote		Promastigote	Amastigote
*Artemisia vulgaris*	460.9 ± 38.5	179.7 ± 15.2	465.2 ± 41.8	1.0	2.6
*Berberis vulgaris*	352.0 ± 28.7	76.8 ± 6.3	357.7 ± 29.3	1.01	4.7
AmB (Control)	0.046 ± 0.0019	0.037 ± 0.0009	13.9 ± 0.6	300	375

*Selectivity index (SI) values were calculated as the ratio of CC₅₀ to IC₅₀ and are therefore derived parameters. Consequently, no post hoc statistical analysis was applied directly to SI values. Statistical analyses were performed on the original IC₅₀ and CC₅₀ datasets as described in the Methods section. IC₅₀: Half-maximal inhibitory concentration. CC₅₀: 50% cytotoxic concentration in THP-1 macrophages. SI (Selectivity Index) = CC₅₀/IC₅₀. Values represent mean ± SD from three independent experiments. AmB: Amphotericin B (Control drug)

**Table 5:** In vitro antitrichomonal activity (IC₅₀), cytotoxicity (CC₅₀), and selectivity index (SI) values of *Artemisia vulgaris* and *Berberis vulgaris* plant extracts against *Trichomonas vaginalis* trophozoites

Extracts	IC_50_ (µg/mL)	CC_50_ (µg/mL)	SI (CC₅₀/IC₅₀)
*Artemisia vulgaris*	104.4 ± 9.2	465.2 ± 38.7	4.5
*Berberis vulgaris*	68.9 ± 6.1	357.7 ± 30.2	5.2
MTZ (Control)	4.35 ± 0.09	43.75 ± 2.05	10.1

*Selectivity index (SI) values are calculated parameters derived from CC₅₀ and IC₅₀ values; therefore, post hoc statistical analysis was not applied directly to SI values. Statistical analyses were conducted on the original IC₅₀ and CC₅₀ datasets as described in the Methods section. IC₅₀: Half-maximal inhibitory concentration. CC₅₀: 50% cytotoxic concentration in THP-1 macrophages. SI (Selectivity Index) = CC₅₀/IC₅₀. Values represent mean ± SD from three independent experiments. MTZ: Metronidazole (Control drug)

## Discussion

Protozoan parasitic infections continue to pose a major global public health challenge, affecting billions of individuals worldwide. According to the World Health Organization, approximately 15 million people are currently infected with *Leishmania* parasites, with two million new cases reported annually and 350 million individuals at risk of exposure. Infections caused by *T. vaginalis* have been associated with adverse pregnancy outcomes, including preterm birth and low birth weight, as well as increased susceptibility to human immunodeficiency virus (HIV) infection and cervical cancer. These data underscore the urgent need for experimental research aimed at identifying parasite-specific therapeutic targets and developing novel, effective treatment strategies [17].

Plants and plant-derived extracts have been used for centuries to treat a wide variety of ailments, ranging from minor conditions such as headaches to severe parasitic infections. Over the past three decades, scientific interest in traditional plant-based remedies has grown considerably, with increasing efforts to evaluate their efficacy and mechanisms of action. Findings from these investigations suggest that plant-derived compounds may be effective in managing bacterial and parasitic diseases, as well as cancer. In particular, experimental studies employing aqueous or alcoholic extracts and essential oils have provided robust evidence for their antiparasitic potential [18, 19]. Notably, between 1981 and 2006, 65% of the 15 antiparasitic drugs approved were derived from natural products or their derivatives. This trend has substantially increased interest in medicinal plants and positioned natural sources as a strategic research domain in drug discovery [20].

The awarding of the 2015 Nobel Prize in Medicine for the discovery of artemisinin from *Artemisia annua* has stimulated growing interest in the pharmacological potential of related species. Among these, *A. vulgaris* stands out due to its wide distribution and long history of traditional use, particularly in gynecological and gastrointestinal disorders. Recent studies have demonstrated diverse biological activities, including antioxidants, hepatoprotective, and antimicrobial effects [21]. In the present study, the ethanolic extract of *A. vulgaris* exhibited dose-dependent antileishmanial activity, inhibiting both promastigote and amastigote forms of *L. major* without cytotoxicity to host macrophages. These findings highlight its selective antiparasitic potential, a result consistent with reports of other *Artemisia* species such as *A. campestris*, *A. aucheri*, and *A. herba-alba* [22]. Phytochemical analyses indicate that *A. vulgaris* is rich in sesquiterpene lactones, flavonoids, and volatile terpenes [23]. Although it does not produce artemisinin, structurally related compounds may act through analogous mechanisms, such as oxidative stress induction and mitochondrial disruption. Indeed, artemisinin itself has been shown to trigger apoptosis-like pathways in *L. donovani*, including mitochondrial depolarization and DNA fragmentation [24]. Flavonoids such as quercetin, abundant in *A. vulgaris*, may further enhance efficacy through enzyme inhibition, redox imbalance, and impairment of parasite energy metabolism [25]. Taken together, the observed antiparasitic activity of *A. vulgaris* likely reflects a synergistic action of its phytochemicals rather than a single compound. While minor contributions from artemisinin-like structures cannot be excluded, the combined effects of sesquiterpene lactones, terpenes, and flavonoids appear to underlie the selective inhibition of *L. major* and *T. vaginalis* observed in vitro. These results strengthen the evidence for *Artemisia* species as promising sources of antiparasitic agents, though further studies are needed to isolate active constituents, validate mechanisms, and assess in vivo efficacy.

When placed in the context of previous research, our findings are broadly consistent with the reported antileishmanial activity of other *Artemisia* species, though some differences in potency are evident. Najm et al. demonstrated that ethanolic extracts of *A. persica* and related Iranian species significantly reduced *L. major* promastigote counts and effectively eliminated intracellular amastigotes in vitro without host cell toxicity, with *A. persica* showing the strongest activity [22]. Similarly, our results with *A. vulgaris* revealed dose-dependent suppression of amastigotes, achieving complete inhibition at 300 µg/mL without impairing macrophage viability, thus supporting the view that *Artemisia*-derived preparations can be both effective and relatively safe. However, the potency of *A. vulgaris* was moderate. Its IC₅₀ against promastigotes (460 µg/mL at 48 h) was comparable to values reported for *A. dracunculus* extracts (IC₅₀ 0.6–0.9 mg/mL after 48–72 h) [26], but weaker than that of *A. sieberi* essential oil, which exhibited strong activity at concentrations as low as 15–20 µg/mL [27]. Such variation underscores the influence of species selection and extraction method on antiparasitic efficacy. Ethanolic macerates, such as that used here, yield a broad spectrum of compounds, including less active polar constituents, whereas essential oils or enriched sesquiterpene fractions may concentrate more potent agents. Future studies should therefore consider fractionation of *A. vulgaris* extracts to determine whether specific nonpolar fractions, such as terpenoid-rich components, display enhanced antileishmanial potency.

Although less extensively investigated than antileishmanial activity, the effects of our plant extracts against *T. vaginalis* warrant consideration. This flagellated protozoan, which relies on hydrogenosomes rather than conventional mitochondria, is the etiological agent of trichomoniasis. Current therapy with metronidazole and other 5-nitroimidazoles remains effective but is increasingly challenged by resistance and adverse effects, including potential carcinogenicity. Consequently, the exploration of herbal remedies and natural products as alternative or complementary antitrichomonal agents has gained momentum, with several *Artemisia* species among those demonstrating promising activity. Previous studies have shown that extracts of *Artemisia* and *Zataria* species can immobilize and lyse *T. vaginalis* trophozoites in vitro, supporting their traditional use in vaginal infections [28]. Consistent with this, the essential oil of *Artemisia ludoviciana* demonstrated a concentration-dependent reduction in trophozoite viability, achieving complete eradication within 48 h at 64 mg/mL [18]. Our findings complement these reports: ethanolic extract of *A. vulgaris* showed dose-dependent antitrichomonal activity, with an IC₅₀ of 104.4 µg/mL. At 50–100 µg/mL, inhibition remained partial, whereas complete lysis was evident at ≥ 200 µg/mL by 48 h. Taken together, these results indicate that *A. vulgaris* ethanolic extract exerts measurable but moderate potency compared to more concentrated essential oils. Nevertheless, the findings support its therapeutic potential and justify further work focusing on fractionation, mechanism elucidation, and optimization of active constituents.

*Berberis vulgaris*, a medicinal plant belonging to the Berberidaceae family, is widely cultivated across Asia and Europe. Its principal bioactive constituent, berberine, is an isoquinoline alkaloid also produced by other species such as *Coptis japonica*, *Coptis spp.*, and *Berberis petiolaris*. Pharmacologically, both *B. vulgaris* and berberine have been associated with a wide range of therapeutic properties, including anti-inflammatory, sedative, antipyretic, antiemetic, antioxidant, anticholinergic, antiarrhythmic, antileishmanial, antimicrobial, and antimalarial activities [29]. Extensive evidence supports the antiparasitic activity of *B. vulgaris* and its alkaloids. Kaneda et al. [30] reported that berberine significantly inhibited the growth of *Entamoeba histolytica*, *Giardia lamblia*, and *T. vaginalis *in vitro, while also inducing morphological alterations. Similarly, berberine derivatives suppressed parasite load in the liver and reduced ulcer size in hamsters infected with *L. donovani* and *L. braziliensis*, performing comparably to meglumine antimoniate [31]. Soffar et al. [32] further demonstrated that berberine exhibited effects equivalent to metronidazole against *T. vaginalis* and suggested its potential as a safer alternative in drug-resistant cases. In the present study, the ethanolic extract of *B. vulgaris* displayed marked in vitro activity against *L. major*, with IC₅₀ values of 352 µg/mL for promastigotes and 76.8 µg/mL for amastigotes, while showing no cytotoxicity toward THP-1 macrophages. Against *T. vaginalis*, the extract yielded an IC₅₀ of 68.9 µg/mL. Although no significant activity was observed at concentrations between 50–62.5 µg/mL during the first 24–48 h, motility reduction became evident at ≥ 62.5 µg/mL, and complete trophozoite lysis occurred at 125 µg/mL. These findings are in line with previous studies confirming the antiparasitic potential of *B. vulgaris*. Comparative studies strengthen this interpretation. Mahmoudvand et al. (2015) showed that *B. vulgaris* extracts exhibited significant in vitro efficacy against *L. tropica* and *L. infantum* amastigotes in infected macrophages, with concentration-dependent effects and limited toxicity to host cells [33]. Similarly, direct comparisons demonstrated that purified berberine inhibited *Leishmania* promastigotes by ~ 70% at 1 mM, while alcoholic extracts of *B. vulgaris* root achieved ~ 90% inhibition at 100 mg/mL [34]. Specifically, comparative anti-leishmanial assays demonstrated that bark extracts exhibited the strongest inhibitory activity against *L. major* promastigotes (IC₅₀ 4.95 ± 0.36 mg/mL), followed by root extracts (IC₅₀ 7.07 ± 0.18 mg/mL) and leaf extracts (IC₅₀ 8.25 ± 0.29 mg/mL). These findings indicate that the bark, known to be particularly rich in berberine, constitutes the primary active source, whereas the leaves display relatively weak activity [35]. In our study, the use of whole aerial parts likely introduced only moderate amounts of berberine, together with other alkaloids, which may account for the intermediate efficacy observed. Importantly, promising in vivo results have also been reported. Topical application of *B. vulgaris* alcoholic extract in a murine model of CL significantly reduced lesion size, with outcomes comparable to standard therapy [36]. Collectively, these findings indicate that the antiparasitic effects observed in vitro can translate to in vivo efficacy and, with appropriate formulation and dosing strategies, may hold clinical relevance.

From the perspective of *Berberis*, direct investigations on *T. vaginalis* remain limited; nevertheless, there is substantive evidence indicating that berberine and its related alkaloids exert antitrichomonal activity. Notably, clinical studies on bacterial vaginosis have demonstrated that *B. vulgaris*, particularly in combination with metronidazole, can enhance therapeutic outcomes, thereby highlighting its broader antimicrobial potential and supporting its consideration as a complementary option in trichomoniasis management [37]. In this context, investigations on other berberine-containing species, such as *Argemone mexicana*, provide further support for this hypothesis. Methanolic extracts of *A. mexicana* stems and leaves exhibited significant inhibitory effects on *T. vaginalis* (IC₅₀ 70.6 and 67.2 μg/mL, respectively), with berberine and jatrorrhizine identified as the most abundant bioactive compounds [38]. In line with these findings, the present study demonstrated that treatment with *B. vulgaris* extract at concentrations ≥ 125 µg/mL resulted in a complete loss of *T. vaginalis* motility and viability within 48 h, a remarkable outcome for a crude extract. The observed antitrichomonal activity (IC₅₀ 69 µg/mL) was slightly less potent than that of metronidazole; however, considering the unrefined nature of the extract, the results are highly promising. Collectively, these data suggest that *B. vulgaris* possesses a clinically relevant antitrichomonal potential which, through fractionation or synergistic interactions among its constituents, could be further optimized. Thus, *B. vulgaris* may represent a valuable complementary or alternative therapeutic option for trichomoniasis, particularly in the context of drug resistance or in populations with a preference for plant-based remedies.

The selectivity index (SI) is a key parameter for evaluating the efficacy of drugs, natural compounds, and plant extracts, and is widely applied in in vitro assays that assess the inhibition of parasite proliferation. SI is calculated as the ratio of the CC₅₀ value for cytotoxic activity to the IC₅₀ value for antileishmanial activity [15]. Various criteria have been proposed to define selective antiparasitic activity in medicinal plant extracts: Tempone et al. [39] suggested SI > 1, Arevalo-Lopez et al. [40] SI > 2, and Joshi et al. [41] SI > 3 as indicative of meaningful selectivity. Within this framework, the SI values of *A. vulgaris* extract were 1.0 for *L. major* promastigotes, 2.6 for *L. major* amastigotes, and 4.5 for *T. vaginalis* trophozoites, while *B. vulgaris* extract exhibited SI values of 1.01, 4.7, and 5.2, respectively. These findings indicate selective activity against protozoa over mammalian cells at the tested concentrations, a profile comparable to many early-stage natural products leads and acceptable for proof-concept in antiparasitic drug discovery. From a safety and selectivity perspective, both extracts therefore appear to act preferentially on protozoa rather than on host cells. Nevertheless, their potency remains modest compared with reference drugs. Metronidazole displayed highly specific activity against anaerobic protozoa, and amphotericin B achieved SI values well into the hundreds owing to nanomolar parasite-killing potency. The comparatively moderate SI values of *A. vulgaris* and *B. vulgaris* underscore the need for careful evaluation, as prolonged exposure to high concentrations (e.g., > 300 µg mL⁻^1^) could begin to affect host cells. Finally, the THP-1 macrophage model employed to estimate cytotoxicity provides only an initial indication of safety; in vivo toxicity may differ. Both *A. vulgaris* and *B. vulgaris* have a long history of traditional medicinal use and, in the case of *B. vulgaris*, culinary consumption, suggesting an encouraging background of tolerability. Even so, rigorous toxicological evaluation in animal models remains essential before any clinical application. Collectively, these observations support *A. vulgaris* and *B. vulgaris* as promising early stage antiparasitic leads, while highlighting the need for further phytochemical purification and comprehensive in vivo safety studies to optimize their therapeutic potential.

Despite the promising antileishmanial and antitrichomonal activities observed in the present study, several limitations should be acknowledged. First, all biological evaluations were conducted exclusively under in vitro and ex vivo conditions. Although these models are widely accepted for preliminary screening, they cannot fully replicate the complexity of host–parasite interactions in vivo. Therefore, the therapeutic efficacy, pharmacokinetics, and safety profiles of the extracts require further validation in appropriate animal models. Second, the study employed crude ethanolic extracts of Artemisia vulgaris and Berberis vulgaris without chemical standardization or fractionation. Consequently, the specific bioactive constituents responsible for the observed antiparasitic effects could not be definitively identified, nor could potential synergistic or antagonistic interactions among phytochemicals be dissected. Advanced phytochemical analyses and bioactivity-guided fractionation will be necessary to clarify the contribution of individual compounds. Finally, mechanistic investigations—such as the evaluation of oxidative stress modulation, nitric oxide (NO) production, mitochondrial dysfunction, or apoptosis-related pathways in the parasites—were beyond the scope of the present work. While previous studies suggest that compounds such as sesquiterpene lactones, flavonoids, and berberine may act through these mechanisms, direct experimental confirmation was not performed here. Similarly, additional biological activities, including antioxidant capacity, were not assessed and warrant future investigation. Taken together, these limitations highlight that the current findings should be interpreted as proof-of-concept evidence supporting the antiprotozoal potential of Artemisia vulgaris and Berberis vulgaris. Further in-depth mechanistic, phytochemical, and in vivo studies are required to fully define their therapeutic applicability.

In conclusion, the present findings demonstrate that *A. vulgaris* and *B. vulgaris* exhibit substantive antileishmanial and antitrichomonal activities in vitro, attributable to phytochemicals such as sesquiterpene lactones, flavonoids, and particularly berberine. These compounds likely act through multiple mechanisms—including induction of oxidative stress, disruption of critical organelles, and inhibition of nucleic acid and protein synthesis—while maintaining relatively low cytotoxicity toward host cells, thereby providing encouraging leads for new antiparasitic therapies. Although the moderate potency of crude extracts precludes their direct therapeutic application, the calculated SI values above unity underscore their selectivity and highlight their value as proof-of-concept candidates in antiparasitic drug discovery. Bridging our experimental results with existing phytochemical knowledge not only addresses previous gaps in mechanistic insight but also strengthens the scientific rationale for their further development. Future research should focus on isolating and quantifying active constituents using advanced analytical techniques (e.g., HPLC, LC–MS), evaluating potential synergistic interactions, and optimizing delivery strategies—such as topical hydrogels or nanofiber dressings for cutaneous leishmaniasis and vaginal gels or suppositories for trichomoniasis. Importantly, their efficacy against drug-resistant strains warrants investigation, given the urgent need for alternative therapies. Collectively, these considerations reinforce the potential of *A. vulgaris* and *B. vulgaris* as valuable sources of plant-derived bioactive compounds and underscore their promise in the development of safe, effective, and innovative treatments for leishmaniasis and trichomoniasis.

## Supplementary Information

Below is the link to the electronic supplementary material.

Supplementary Material 1

## Data Availability

Data will be made available on request.
