# Supplementary material for: Antiprotozoal Activity of Artemisia vulgaris and Berberis vulgaris Against Leishmania major and Trichomonas vaginalis
Source: Acta Parasitol. 2026 Feb 16;71(1):44. doi: 10.1007/s11686-026-01224-1 (PMC12909463; doi:10.1007/s11686-026-01224-1)
Supplement: Supplementary file 1 — Supplementary Material 1 [file 11686_2026_1224_MOESM1_ESM.docx]

**Cover letter**

12/11/2025

**The Editorial Office of *Acta Parasitologica***

**Dear Editor,**

I am pleased to submit our manuscript, " **Antiprotozoal Activity of *Artemisia vulgaris* and *Berberis vulgaris* Against *Leishmania major* and *Trichomonas vaginalis***", for consideration in *Acta Parasitologica*. Protozoan parasitic infections, including leishmaniasis and trichomoniasis, represent a significant global health challenge, affecting millions and contributing to the burden of infectious diseases. Our study examines the *in vitro* and *ex vivo* antiparasitic potential of *A. vulgaris* and *B. vulgaris* plant extracts, focusing on their effects against *L. major* promastigotes and amastigotes, as well as *T. vaginalis* trophozoites.

We believe this research is particularly relevant to your journal’s readership, given its emphasis on identifying natural product-based therapeutic agents with favorable selectivity profiles and low cytotoxicity. By leveraging standardized *in vitro* assays, microscopic *ex vivo* techniques, and rigorous cytotoxicity and selectivity index calculations, our findings contribute to the growing body of evidence that plant-derived compounds may serve as valuable alternatives or adjuncts to existing antiparasitic treatments.

*Acta Parasitologica*’s commitment to advancing parasitology research makes it an ideal venue for our work. Our findings are not only aligned with the journal’s focus on innovative approaches to parasitic diseases but also provide a platform for further exploration of bioactive compounds from natural sources. We hope that this study will inspire additional research into the development of new antiparasitic therapies and encourage interdisciplinary efforts within the parasitology community.

We confirm that this manuscript has not been published elsewhere and is not under consideration by any other publication. All authors have read and approved the final manuscript and have no conflicts of interest to declare.

Thank you for considering our submission. We look forward to the possibility of contributing to your esteemed journal and are happy to provide any additional information or materials that might be helpful during the review process.

We have no conflicts of interest to disclose.

Please address all correspondance concerning this manuscript to me at ([tulay.aksoy@inonu.edu.tr](mailto:tulay.aksoy@inonu.edu.tr) ).

Thank you for your consideration of this manuscript.

Sincerely,

| **Corresponding Author** |  |
| --- | --- |
| **Dr. Tülay AKSOY**  Medical Parasitology Department  Medical School  Inonu University  Malatya, Türkiye | **Prof. Dr. Nogay GİRGİNKARDEŞLER**  Medical Parasitology Department  Medical School  Manisa Celal Bayar University  Manisa, Türkiye |
|  |  |
|  |  |
| **Prof. Dr. İ. Cüneyt BALCIOĞLU**  Medical Parasitology Department  Medical School  Manisa Celal Bayar University  Manisa, Türkiye | **Prof. Dr. Ali A. KİLİMCİOĞLU**  Medical Parasitology Department  Medical School  Manisa Celal Bayar University  Manisa, Türkiye |
